# High-Flow Nasal Aerosol Therapy; Regional Aerosol Deposition and Airway Responsiveness

**DOI:** 10.1089/jamp.2024.0026

**Published:** 2024-12-02

**Authors:** Srinivasa Potla, Gerald C. Smaldone

**Affiliations:** Division of Pulmonary, Critical Care and Sleep Medicine, Department of Medicine, Stony Brook University Medical Center, Stony Brook, New York, USA.

**Keywords:** aerosol deposition, breath-enhanced jet nebulizer, high-flow nasal cannula

## Abstract

**Introduction:**

In normal subjects, during tidal breathing, aerosols deposit by settling in small airways. With obstructive lung disease (OLD), collapse of airways during expiration causes turbulence and increased deposition in central airways. High-flow nasal cannula (HFNC) therapy, washing out dead space, may affect deposition mechanisms and drug delivery. This study compared aerosol deposition and airway responsiveness in OLD after traditional and HFNC nebulization therapy.

**Methods:**

Twelve subjects with moderate to severe OLD participated in a two-day study. Spirometry was measured pre- and post-aerosol inhalation. On Day 1 (D1) subjects tidally inhaled radiolabeled albuterol (^99m^Tc DTPA) by mouth via AeroTech II, (Biodex. Shirley, NY). Day 2 (D2) inhalation was via HFNC using *i-AIRE* (InspiRx, Inc. Somerset, NJ). The HFNC system (60 L/m) was infused by syringe pump at 50 mL/h. D2 lung deposition was monitored in real time by gamma camera to match D1. Pre and post heart rate, O_2_ sat, and nasopharyngeal deposition (NP) were measured. Mechanistic contributions were modeled using multiple linear regression (MLR) of deposition rate (DR µg/m) as a function of breathing frequency, airway geometry (FEV_1_), and parenchymal integrity (DLCO).

**Results:**

Albuterol lung depositions were matched (*p* = 0.13) with D1 central/peripheral (sC/P) ratios 1.99 ± 0.98. Following HFNC, peripheral deposition increased (31% ± 33%, sC/P = 1.51 ± 0.43, *p* = 0.01). D2/D1% change FVC increased by 16.1 ± 16.7% (*p* = 0.003). NP deposition averaged 333% of lung. Heart rate and O_2_ sat were unaffected (*p* = 0.31, *p* = 0.63 respectively). DR analysis was markedly different between D1 (*R*^2^ = 0.82) and D2 (*R*^2^ = 0.12).

**Conclusion:**

In subjects with OLD, HFNC nebulization at 60 L/min was well tolerated and increased peripheral drug delivery. Spirometry significantly improved. Systemic effects were undetected indicating limited nasal absorption. MLR demonstrated that different mechanisms of deposition govern traditional vs HFNC aerosol delivery. Breath-enhanced nebulization via HFNC may provide controllable and effective aerosol therapy in OLD.

## Introduction 

Hypoxemic respiratory failure is often managed with high-flow nasal cannula (HFNC) oxygen therapy.^1^ Besides increasing FiO_2_, the HFNC increases dead space ventilation washing out central airways^2^ and affords the possibility of simultaneous aerosol therapy, introducing nebulized drug particles directly into the oxygen stream.^3,4^ Therefore, the particles may be transported automatically throughout the large airways potentially deep into the lungs. Traditional aerosol therapy, in acutely ill patients, often uses nebulizers ventilated with tidal breathing. From the nebulizer, only the inhaled tidal gas contains particles, which must penetrate the dead space, then depositing chiefly by settling in distal airways. This process depends on particle size, small airway geometry, and breathing pattern, primarily inspiratory phenomena.^5^ Patients with obstructive lung disease (OLD) can have an additional mechanism of deposition. These patients, during expiration, may breathe with the tidal loop superimposed on their expiratory flow volume curve resulting in central airway narrowing. It is known that changes in central airway geometry, during expiration, are associated with turbulent expiratory deposition of particles at sites of flow limitation.^6^

Theoretically, this analysis suggests that transport of inhaled particles throughout the airways may be very different depending on the path of particle delivery e.g., via the mouth or the nose. Therefore, different mechanisms of deposition, particularly in obstructed patients, may affect different airways. The purpose of the present study was to define sites of deposition of radiolabeled albuterol aerosols in obstructed patients using a protocol designed to compare traditional to HFNC delivery. To simplify this analysis, particle size and total lung deposition were controlled. Spirometry provided an index of airway geometry. Parenchymal integrity was estimated by diffusion capacity (DLCO). Besides measuring total and regional aerosol delivery, using an active drug (albuterol) facilitated an estimate of potential differences in efficacy between these forms of therapy, and, of practical value, an assessment of potential side effects from systemic nasal absorption. Our goal was to set the stage for clinical trials of aerosol therapy using HFNC delivery.

## Methods

### Principles

To compare sites of deposition to responsiveness, we controlled total lung dose after both tidal and HFNC aerosol delivery using known aerosols matched by mass median aerodynamic diameter (MMAD), geometric standard deviation (GSD), and with published aerosol distributions containing less than 2% of the mass at 5 µm or above. On the first day (D1) the subject inhaled albuterol using a jet nebulizer (AeroTech II, Biodex. Shirley, NY, MMAD ± GSD 1.2 ± 2.5 µm^7–10^), well-characterized by our group, known to produce small particles during tidal breathing. This inhalation defined the D1 lung dose. On the second day (D2), by monitoring the gamma camera, the same lung dose of albuterol was deposited via HFNC delivery. The nebulizer used during high flow experiments (*i-AIRE*; InspiRx, Inc. Somerset, NJ, MMAD ± GSD 1.1 ± 1.4 µm^11^) is a newly developed breath-enhanced nebulizer that uses the high gas flow supplied to the patient to increase aerosol production. The increased production of aerosol overcomes the losses seen with conventional nebulizers throughout the high-flow circuit. In recent studies on the bench^11^ and in normal volunteers,^12^ this nebulizer was shown to deliver aerosols via the nasal catheter at high flow (60 L/m). Using this device, dose to the lung at these gas flow rates can be regulated using continuous infusion. On each day, drug efficacy was compared using pre and post spirometry. Possible drug side effects were assessed by monitoring heart rate and oxygen saturation before and after therapy.

### Day 1 protocol

Twelve subjects with moderate-to-severe OLD were enrolled after signing the Stony Brook IRB-approved consent form for the two-day study. Adult subjects were eligible if they had moderate to severe OLD (known from our clinical practice) and a history of using nebulized albuterol. Subjects were ineligible if they had documented or diagnosed restrictive lung disease, significant comorbidities that would make testing unsafe (severe heart failure with reduced ejection fraction, etc.), and/or uncontrolled coughing. Before beginning aerosol inhalation, 2500 µg of Albuterol was placed into an AeroTech II mixed with approximately 3 mCi of 99mTechnetium bound to Diethylenetriamine pentaacetate (^99m^Tc DTPA). The measured activity defined the nebulizer charge and zero time (T = 0). The nebulizer, in a lead container, was connected to a Y-piece attached to a mouthpiece and expiratory filter. We assumed that ^99m^Tc DTPA, which was homogeneously distributed in the albuterol solution and nebulized particles, served as a direct marker of the drug.^13,14^ After baseline spirometry, subjects were seated in front of a gamma camera (Maxi Camera 400; General Electric, Horsholm, Denmark; Model 604/150/D; Power Computing, Austin TX; Nuclear Power, version 3.0.7; Scientific Imaging, Inc., Thousand Oaks, CA, USA). First, a 15-minute background image was obtained. Camera efficiency was measured using a filter paper labeled with 300–500 µCi scanned on the camera face. Then, a double-walled Lucite container was placed in front of the subject’s chest. The container, with a space between the walls, was filled with normal saline instilled with approximately 2 mCi of radioactivity diffused throughout. A two-minute transmission image was taken to aid in determining optimal position of the subject and estimate regional lung volumes. Baseline heart rate and oxygen saturation were obtained.

After baseline imaging, subjects, using tidal breathing, inhaled radiolabeled aerosol from the nebulizer with relaxed tidal breathing. The nebulizer was driven with air at 50 PSIG and a flow of 10L/min. The treatment was given until completion (about 8 min). The total number of treatment breaths and time of delivery were recorded (breaths/min). After completing nebulizer treatment, post treatment deposition images (1 min), vitals, and spirometry were obtained.

Using the transmission scan, regions of interest were drawn over the lungs.^15,16^ Regional counts were background and decay corrected to T = 0. To prepare for the D2 experiment, albuterol deposition in the lungs was calculated using eq. (1). The basis of eq. (1) is 1 minute.

(1) D1 depo = (A (AF)/E) (specific nebulizer charge)

D1 depo (µg albuterol deposited in the lung regions) was calculated by converting measured counts to µg. As shown in eq. (1), A, activity in the lung regions (cts) was corrected for chest wall attenuation using the equation of Fleming et al.^12,17,18^ which defined AF. The corrected counts were converted to µCi using the measured camera efficiency (E cts/µCi). Multiplying by the specific activity of the nebulizer charge (µg/µCi) resulted in µg albuterol deposited in the lungs.

### Day 2 protocol

The goal of the D2 protocol was to match the lung albuterol deposition defined on D1. This was carried out by monitoring gamma camera activity during aerosol delivery until the appropriate counts were obtained to reflect the albuterol lung deposition from D1. On D2, the albuterol test solution consisted of 6–8 mL of albuterol stock solution (Nephron. West Columbia, SC.) (5 mg/mL) mixed with approx. 8 mL of normal saline and 30 mCi ^99m^Tc DTPA. Measurement of syringe activity (µCi) was defined at T = 0. The expected cts/min on D2 at T = 0, was calculated using eq. 2. The basis of eq. (2) was one minute.

(2) D2 regional cts (@T=0) = (D1 depo/specific syringe charge) CF

D1 depo (µg albuterol) was divided by the D2 specific syringe charge (µCi syringe/total µg syringe albuterol) to yield the µCi desired on D2 in the lungs at T = 0. D2 cts were calculated by multiplying by a conversion factor CF (cts/µCi) measured for each subject on D1.

The D2 cts expected from eq. (2) would decline due to radioactive decay from T = 0 to the actual time of scanning (approx. 1 hour or a decay of ∼10%) requiring real time correction (eq. 3). A final correction estimating scatter between imaging cts on the whole camera field and regional lung cts (approximately 30%) was applied to eq. (3) which accounted for activity outside of the regions.

(3) D2 cts (whole lung, real time) = (D2 cts @T=0) (0.9) (1.3)

After the preliminary calculations estimating real time whole lung D2 cts, the subject was fitted with a humidified HFNC (Optiflow, Fisher and Paykel, Auckland, New Zealand). The catheter was interfaced with the i-*AIRE* nebulizer and an Alaris Infusion Pump (Alaris Pump Module, Becton, Dickinson and Co., Franklin Lakes, NJ, USA) as previously described by Jayakumaran et al.^12^ On one side, the pump was outfitted with a 1L IV bag of nonradioactive normal saline. On the other side, an infusion syringe containing the radiolabeled test solution. First, subjects were trained on the HFNC system by incrementally up titrating air flow from 20 L/min to 60 L/min while simultaneously infusing nonradioactive normal saline into the nebulizer at rates of 20 mL/h to a goal of 50 mL/h. After the subject was comfortable on the test air flow (60 L/min) and infusion rate (50 mL/h), the normal saline infusion was stopped, and the radiolabeled albuterol infusion was started at 50 mL/h. The patient was continuously monitored by the gamma camera with serial 1-minute scans until the whole lung count rate approximated that expected from eq. (3). When goal activity was reached, infusion and air flow were stopped, and the nasal cannula removed. Post treatment images of the lungs and a lateral scan of the head measuring nasopharyngeal deposition were obtained. Post treatment vitals and spirometry were measured.

### Analysis

Regional analysis was performed on whole lung and central regions as described by Samuel et al.^15^ and Smaldone et al.^16^ The central region contained approximately one third of the whole lung to encompass the central airways. Deposition regions were normalized for lung volume using the transmission image ratio (Tc/P) resulting in regional deposition corrected for regional lung volume (Sc/P).

The rate of albuterol deposition (DR [µgm albuterol deposited/m]) was estimated by dividing deposited albuterol in the lungs by the time of inhalation as shown in eq. (4).

(4) DR = D1 or D2 deposition/treatment time

Statistical analysis was performed using GraphPad Prism for Mac OS X (GraphPad Software version 10.2.2, San Diego, CA, USA). Data are reported as mean ± SD. Paired data were analyzed using the Wilcoxon matched pairs signed rank test.

Mechanistic factors thought to affect deposition were assessed using multiple linear regression (MLR). We tested a model that measured D1 and D2 deposition rates (as a function of particle residence time [breathing rate (breaths/min)], airway geometry (forced expiratory volume in one second), percent predicted (FEV_1_%), and parenchymal integrity (DLCO%). The model is illustrated by eq. (5).

(5) $$\text{DR ∼ intercept+}\beta_{1}\text{Breaths+}\beta_{2}\text{preFEV1\% +}\beta_{3}\text{DLCO\%}$$

The multiple regression analysis was an attempt to see if the sum of factors thought to govern DR explained variability in measured values. This analysis indicates the relative importance of each variable to the overall correlation.

## Results

Baseline demographics, anthropomorphic details, and spirometry data of the test subjects are listed in Table 1 as well as the spirometric responses to albuterol on D1and D2. All subjects had abnormal FEV_1_/FVC ratios. Most had reduced DLCO suggesting parenchymal disease. Changes in FVC were significantly greater following HFNC delivery compared with traditional nebulization (D1 FVC increased 10% ± 12.0% D2, 26.1% ± 19.9%, *p* = 0.003).

**Table 1.** Baseline Demographics and Spirometry with Change in Spirometry D1 & D2

Patient ID	Sex	Age	FEV_1_ (%)	FVC (%)	FEV_1_/FVC	D1 FEV1 Δ%	D1 FVC Δ%	D2 FEV1 Δ%	D2 FVC Δ%	DLCO (%)
1	M	75	56	89	0.48	28.0	25.7	45.1	69.4	87
2	F	52	30	67	0.37	9.5	4.7	53.2	0.0	92
3	F	64	30	55	0.42	1.8	8.9	5.0	18.3	38
4	F	64	35	72	0.38	1.3	1.0	10.3	32.1	43
5	M	94	41	54	0.55	30.4	39.4	18.3	38.9	50
6	M	69	41	64	0.48	3.5	2.0	8.2	13.9	51
7	F	59	72	105	0.53	1.1	−0.3	12.7	22.5	38
8	M	78	37	66	0.43	2.8	8.9	6.7	16.3	55
9	M	76	55	78	0.54	13.0	0.3	6.3	8.0	40
10	M	77	38	55	0.53	13.2	9.3	21.9	55.2	58
11	M	43	68	99	0.56	18.3	2.9	28.0	20.2	102
12	M	56	57	77	0.59	27.4	17.0	23.5	17.9	79
Mean ± SD	—	67 ± 14	47 ± 14	73 ± 17	0.49 ± 0.07	12.5 ± 11.2	10.0 ± 12.0	19.9 ± 15.6	26.1 ± 19.9^*^	61 ± 23

% = % predicted.

Δ% = % change of spirometric values.

^*^ *p* = 0.003 compared with D1 FVC Δ%.

FEV_1_%, forced expiratory volume in one second, percent predicted; SD, standard deviation.

**FIG. 1.**
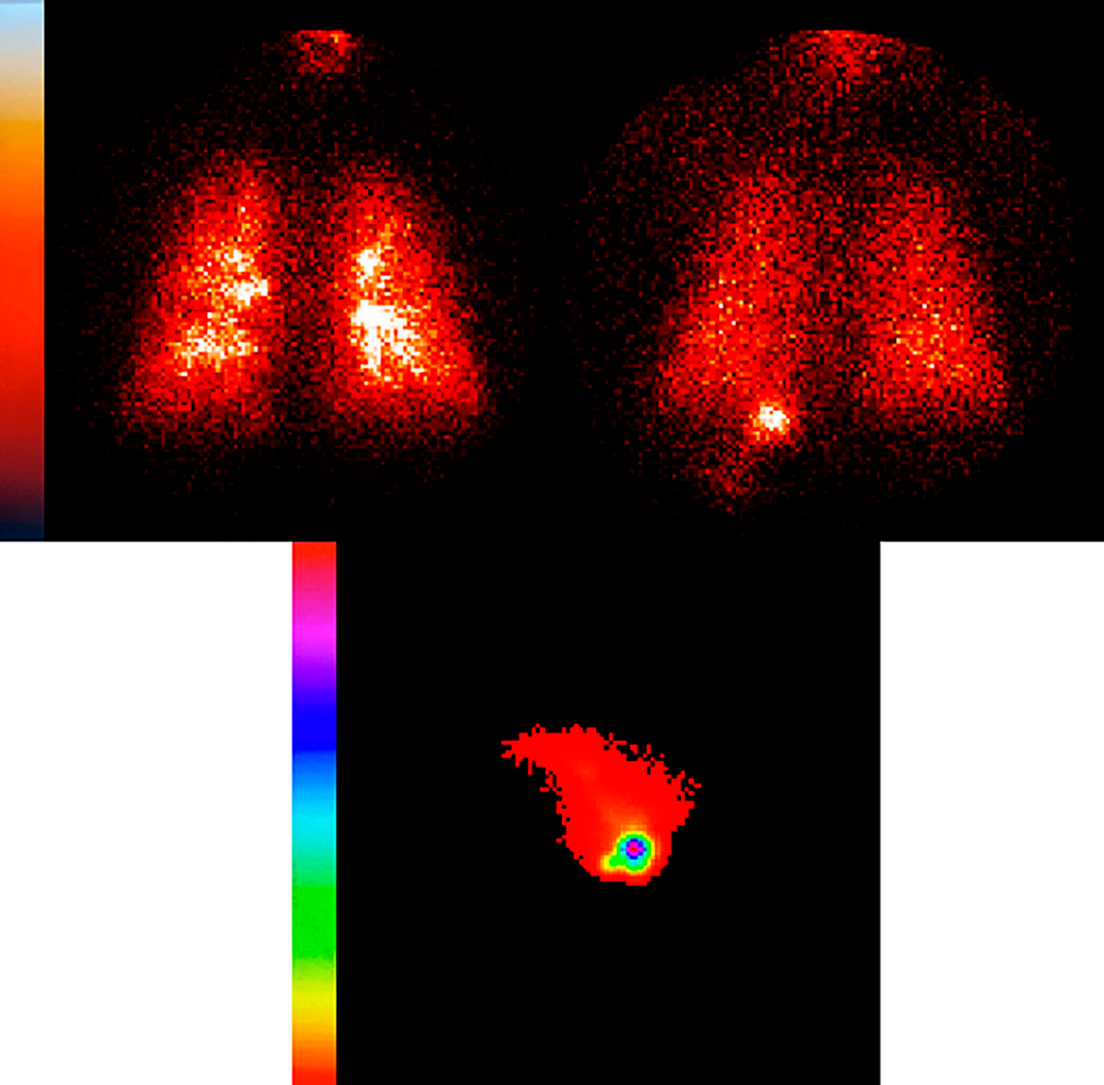
Deposition images of Subject 11. *Top left*: D1 lung deposition sC/P 1.85. *Top right*: D2 sC/P 1.57. *Bottom*: Nasal deposition, subject was facing to the left. Left and right reversed on gamma camera. Camera spectrum shown.

Characteristic deposition images are shown in Figure 1 (subject 11). On D1 (left), the visual pattern reveals the central airways. For the same subject, on D2, deposition on the right appears more uniform (D1 sC/P 1.85, D2 1.57). In addition, the lateral nasal D2 deposition image is shown suggesting deposition is mostly in the nasal vestibule.

**FIG. 2.**
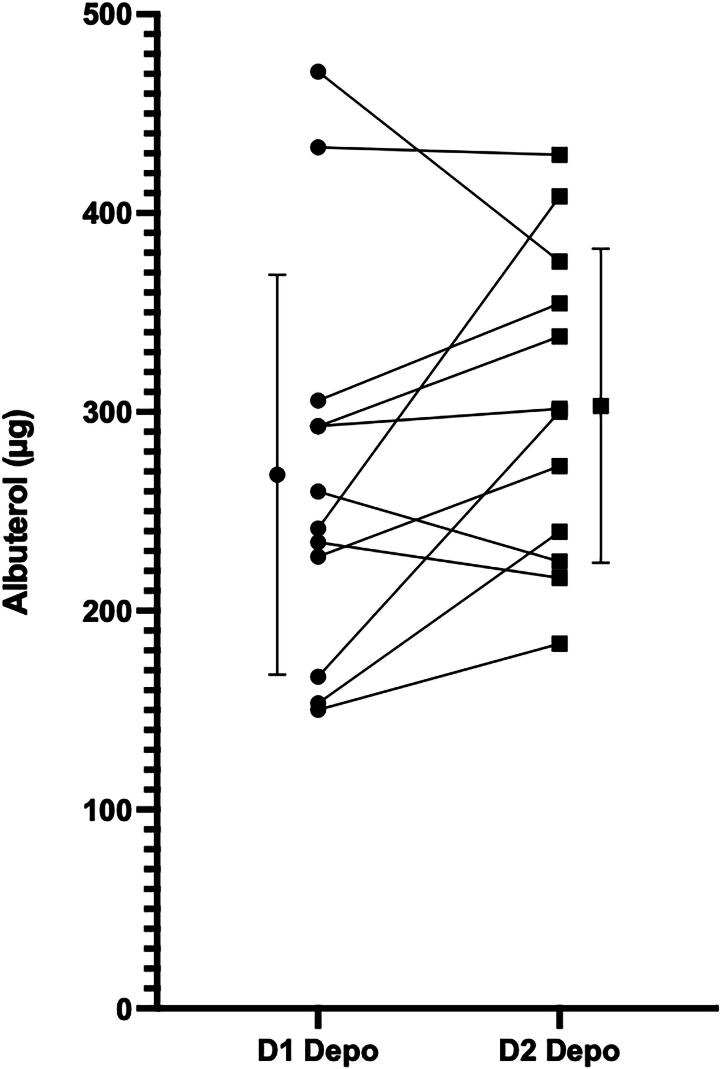
Lung dose of albuterol. D1 deposition 269.05 µg ± 100.86, D2 303.75 µg ± 79.23 (*p* = 0.13).

D1 and D2 whole lung depositions are plotted on Figure 2 and tabulated in Table 2. As shown in the figure, there was considerable scatter between subjects but mean values of D1 and D2 depositions were closely matched. Mean deposition on D1 with traditional nebulization was 269 µg albuterol ± 101 (mean ± SD), and on D2, with use of HFNC, 304 µg albuterol ± 79 (*p* = 0.13).

**Table 2.** Central to Peripheral Ratios: Aerosol (aC/P), Transmission Image (tC/P), Normalized (sC/P) and Deposition

Subject ID	D1 aC/P	D1 tC/P	D1 sC/P	D2 aC/P	D2 tC/P	D2 sC/P	D1 lung depo (µg)	D2 lung depo (µg)	D2 nasal depo (µg)
1	1.31	0.67	1.98	1.06	0.64	1.69	471	376	1358
2	1.24	0.51	2.46	0.74	0.55	1.36	227	273	1736
3	3.11	0.74	4.21	1.64	0.75	2.19	235	217	1135
4	2.38	0.72	3.36	1.79	0.76	2.36	167	300	554
5	1.60	0.68	2.31	1.13	0.68	1.66	241	408	1364
6	1.00	1.02	1.01	1.07	1.13	0.95	154	240	1131
7	0.71	0.63	1.11	0.68	0.60	1.14	150	183	562
8	1.13	0.81	1.39	1.02	0.73	1.41	260	225	579
9	0.89	0.74	1.20	0.73	0.78	0.95	293	302	1422
10	1.13	0.63	1.82	0.76	0.58	1.36	306	355	811
11	1.26	0.70	1.85	1.03	0.66	1.57	433	429	2485
12	0.77	0.65	1.18	0.87	0.62	1.45	293	338	622
Mean ± SD	1.38 ± 0.70	0.71 ± 0.12	1.99 ± 0.98	1.04 ± 0.35	0.70 ± 0.15	1.51 ± 0.43^*^	269 ± 101	304 ± 79	1147 ± 581

^*^ *p* = 0.01 when compared with D1 sC/P.

**FIG. 3.**
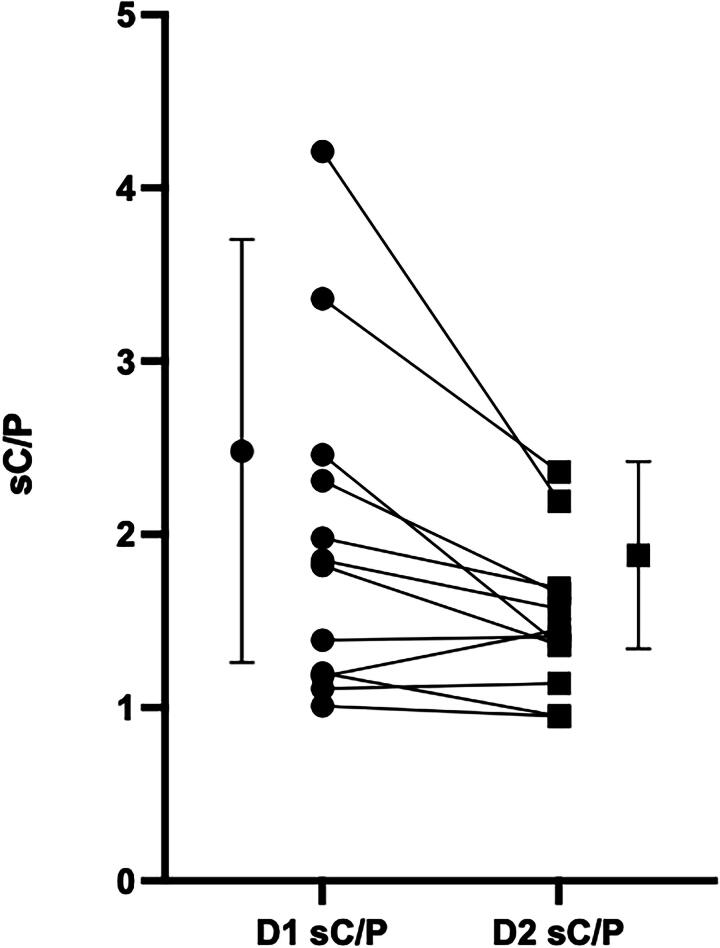
sC/P ratio D1 vs D2. sC/P= ratio of counts in central **(C)** and peripheral **(P)** regions normalized for regional volume determined by transmission scan. D1 1.99 ± 0.98, D2 1.51 ± 0.43. Increase in peripheral penetration 31% ± 33%. *p* = 0.01.

Table 2 also lists values for regional lung deposition. On D1, sC/P averaged 1.99 ± 0.98, declining significantly on D2 to 1.51 ± 0.43 representing an increase of 31% ± 33% in peripheral penetration (*p* = 0.01). Trends are shown in Figure 3. Inspection of the figure indicates that the major shifts in deposition pattern occur for those subjects with the most central deposition on D1.

**FIG. 4.**
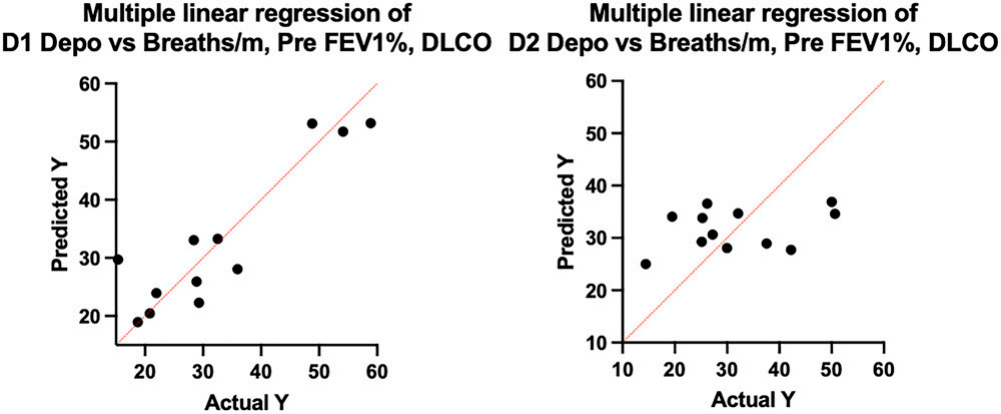
Multiple linear regression: Y axis, DR term predicted from eq. (5), X axis, DR term calculated from data. D1 (*left*) and D2 (*right*) as a function of breaths/min, Pre FEV_1_%, and DLCO%. D1 *R*^2^ = 0.82. D2 *R*^2^ = 0.12. β coefficients are listed in Table 3.

Multiple linear regression of the parameters thought to govern deposition are shown in Figure 4. Values predicted by the model illustrated in eq. (5) are on the vertical axis plotted against the calculated values on the abscissa. On the left, deposition rate (DR [µg albuterol/m]) measured on D1 appears to be closely estimated by terms related to residence time (breathing rate), airway geometry (FEV_1_%), and parenchymal integrity (DLCO%) with a regression coefficient *R*^2^ = 0.82. All three variables were significant as shown in Table 3. On the right, D2 data for the same parameters indicate a poor correlation, *R*^2^ = 0.12, and no variable was significant. The model suggests that different factors control the DR during HFNC delivery. Individual coefficients for the model are listed in Table 3. Listed are the changes in *R*^2^ (Δ*R*^2^) that reflect the contributions of each variable to variation in DR. For example, DLCO and FEV_1_ contributed equally about 36% of the variability, with breathing rate 10%. This relationship changed markedly with HFNC aerosol delivery with no variable having predictive value.

**Table 3.** Multiple Linear Regression: Drug Deposition Rate (DR) on Day1 and Day2 as a Function of Breaths/Min, Pre FEV1%, and DLCO%

Dependent variables	Independent variables	ß	Standard error	95% CI	*P*	*R* ^2^	Δ*R*^2^
DR D1						0.82	
	Breaths/min	−1.83	0.63	−3.27 to −0.39	0.02		0.10
	Pre FEV1%	0.48	0.17	0.09 to 0.87	0.02		0.37
	DLCO	0.39	0.10	0.14 to 0.58	0.004		0.36
DR D2						0.12	
	Breaths/min	0.77	0.75	−0.968 to 2.51	0.34		0.11
	Pre FEV1%	0.03	0.28	−0.613 to 0.675	0.92		0.002
	DLCO	0.02	0.17	−0.386 to 0.415	0.93		0.001

Regression model: DR ∼ Intercept + β_1_ Breaths/min + β Pre FEV1 % + β_3_ DLCO%.

β_1−3_ slope coefficients.

Δ*R*^2^ contribution of individual variable to the model.

DR, deposition rate.

Nasal deposition of albuterol was three to six times greater in the nose compared with the lungs (Table 2). On average, nose and lung doses for day 2 were 1147 ± 581 µg and 304 ± 79 µg, respectively (*p* = 0.0005). Despite the magnitude of D2, nasal deposition, heart rate, and O_2_ saturation were unaffected. Heart rate and O_2_ saturation measurements are listed in Table 4. The mean changes following aerosol D1 vs D2 heart rate 3.25 beats/min ± 12.8 vs 7.08 beats/min ± 6.68 (*p* = 0.31) saturation −0.75% ± 1.91% vs −0.92 ± 1.51 (*p* = 0.63).

**Table 4.** Heart Rate and Pulse Oximetry

Patient ID	D1 pre HR	D1 post HR	Δ D1 HR	D2 pre HR	D2 post HR	Δ D2 HR	D1 pre SpO2	D1 post SpO2	Δ D1 SpO2	D2 pre SpO2	D2 post SpO2	Δ D2 SpO2
1	64	64	0	75	76	1	98	98	0	98	98	0
2	91	63	−28	101	110	9	98	98	0	99	99	0
3	115	112	−3	96	97	1	98	94	−4	97	94	−3
4	78	101	23	87	87	0	97	97	0	97	97	0
5	76	90	14	68	81	13	96	99	3	97	98	1
6	95	97	2	102	102	0	95	93	−2	95	92	−3
7	70	89	19	73	88	15	99	98	−1	99	96	−3
8	80	81	1	86	85	−1	97	97	0	98	97	−1
9	95	98	3	105	112	7	97	93	−4	93	92	−1
10	78	78	0	69	78	9	95	94	−1	96	94	−2
11	65	72	7	72	86	14	99	99	0	98	98	0
12	60	61	1	63	80	17	98	98	0	98	98	0
Mean ± SD			3.25 ± 12.8			7.08 ± 6.68^*^			−0.75% ± 1.91%			−0.92 ± 1.51%^**^

^*^ *p* = 0.31 when compared with Δ D1 HR.

^**^ *p* = 0.63 when compared with Δ D1 SpO2.

SD, standard deviation.

## Discussion

The present study demonstrates that, in obstructed patients, regional aerosol delivery during HFNC therapy was significantly different than delivery during mouth breathing using a traditional nebulizer. There was a shift in deposition from central to peripheral airways. As shown in Figure 3, shifts in deposition pattern seem to be greater for those patients with the most central D1 deposition.

It is well known that lung deposition during tidal breathing is a function of several patient-dependent factors primarily the breathing pattern and the patient’s airway geometry.^19^ The breathing pattern defines the penetration of tidal air into the dead space as well as the residence time of particles in small airways. These factors vary from patient to patient and, after filling the nebulizer, one therefore cannot predict the lung dose. Our own data measured on D1 in Figure 2 is an example of this variability.

In severe airway obstruction, deposition mechanisms often favor sites in central airways. For example, in emphysema, particles inhaled during tidal breathing pass through the large airways during inhalation but do not deposit because the airways are too large for settling. As they pass further into the lungs, particles may not deposit in the periphery failing to reach distant airways due to increased dead space plus the airways may be pathologically enlarged. Therefore, small particles remain in the airstream and, during expiration, are exposed to local turbulent forces created by choke points in compressed central airways.^6,20^ High-flow therapy may affect deposition in these patients in several ways; for example, transporting entrained particles deeper into the lung as well as interfering with the forces in central airways causing choke point deposition, possibly due to increased intraluminal pressures during HFNC therapy. Consistent with this hypothesis, MLR of macroscopic parameters known to affect deposition during tidal breathing predicted our data only for nebulizer therapy on D1. D2 deposition did not correlate with any of the test variables and appears independent of breathing pattern. The first observation of the latter phenomenon was made by Jayakumaran et al. in normal subjects.^12^ They found that DR was a function primarily of the nebulizer infusion rate and not the breathing pattern.

Albuterol, our test aerosol, has biological effects. While our subjects were not clinically asthmatic, with limited baseline changes following bronchodilators, our biological observations were consistent with the measured changes in regional deposition. Although whole lung deposition matched, changes in small airways function (FVC) were more significant on D2.

Nasal delivery of aerosol to the lungs may be more convenient and more effective, but nasal deposition can be a complicating factor because far more aerosol was deposited in the nose than in the lungs. However, our data indicate this concern is not real for albuterol delivery at these flow rates because particles deposited mostly in the proximal nasal cavity where drug was not absorbed. Nasal deposition studies, focusing on nasal sprays tailored to treat different nasal compartments, have classically reported deposition as a function of particle size, plume geometry, plume velocity, and inspiratory flow rate.^5^ During HFNC aerosol delivery, different from nasal sprays, inspiratory flow rates may dominate local delivery resulting in deposition in the anterior compartments of the nose. Sites of nasal deposition may vary if inspiratory flows are decreased.

The present study was possible because of the increased aerosol output of the breath-enhanced i-*AIRE* nebulizer. At the highest HFNC flow rates, traditional nebulizers fail to deliver significant aerosol to the lungs.^11^ Previous investigators advocated lowering the nasal gas flow to reduce losses and increase delivery.^21^ However, this approach is inconsistent with the clinical requirement of increased flow, needed for oxygenation in the critically ill.

These findings complement the previous study by Jayakumaran et al. Unlike traditional nebulizer bolus aerosol therapy, with unpredictable deposition and response in a given patient, breath-enhanced HFNC delivery allows a health worker to control delivery to the lungs, titrating therapy to a clinical effect while monitoring for side effects. Albuterol represents a short-acting drug amenable to continuous infusion. Prostacyclin, another short-acting drug, can be used in hypoxic patients with acute respiratory distress syndrome. Although clinical trials are needed to further define the potential of this approach, our study shows that HFNC delivery is possible at the highest flows used in the critically ill.

## Conclusion

In subjects with airway obstruction, aerosol deposition in the lungs following HFNC nebulization is more homogenous compared with traditional nebulization. For albuterol, HFNC delivery is safe and may be more effective. Future studies will further define the utility of HFNC nebulization in treating hospitalized patients requiring HFNC oxygen.

## References

[1] Spoletini G, Alotaibi M, Blasi F, et al. Heated humidified high-flow nasal oxygen in adults: Mechanisms of action and clinical implications. Chest 2015;148(1):253–261; doi: 10.1378/chest.14-2871 25742321

[2] Nishimura M. High-flow nasal cannula oxygen therapy in adults: Physiological benefits, indication, clinical benefits, and adverse effects. Respir Care 2016;61(4):529–541; doi: 10.4187/respcare.04577 27016353

[3] Beuvon C, Coudroy R, Bardin J, et al. Beta agonist delivery by high-flow nasal cannula during COPD exacerbation. Respir Care 2022;67(1):9–15; doi: 10.4187/respcare.09242 34702767

[4] Colaianni-Alfonso N, MacLoughlin R, Espada A, et al. Delivery of aerosolized bronchodilators by high-flow nasal cannula during COPD exacerbation. Respir Care 2023;68(6):721–726; doi: 10.4187/respcare.10614 37041023 PMC10209003

[5] Cheng YS. Mechanisms of pharmaceutical aerosol deposition in the respiratory tract. AAPS PharmSciTech 2014;15(3):630–640; doi: 10.1208/s12249-014-0092-0 24563174 PMC4037474

[6] Smaldone GC, Messina MS. Flow limitation, cough, and patterns of aerosol deposition in humans. J Appl Physiol 1985;59(2):515–520. 4030604 10.1152/jappl.1985.59.2.515

[7] Kanth PM, Alaienia C, Smaldone GC. Nebulized mannitol, particle distribution, and cough in idiopathic pulmonary fibrosis. Respir Care 2018;63(11):1407–1412; doi: 10.4187/respcare.06153 30154129

[8] Sagalla RB, Smaldone GC. Capturing the efficiency of vibrating mesh nebulizers: Minimizing upper airway deposition. J Aerosol Med Pulm Drug Deliv 2014;27(5):341–348; doi: 10.1089/jamp.2014.1152 25105472

[9] Smaldone G, Solomita M. Predicting in vivo deposition in vitro. J Aerosol Med Pulm Drug Deliv 2009;22(1):9–10; doi: 10.1089/jamp.2008.0738 19392584

[10] Smaldone GC, Perry RJ, Deutsch DG. Characteristics of nebulizers used in the treatment of AIDS-related pneumocystis carinii pneumonia. J Aerosol Med 1988;1(2):113–126.

[11] Moon J, McPeck M, Jayakumaran J, et al. Enhanced aerosol delivery during high-flow nasal cannula therapy. Respir Care 2023;68(9):1221–1228; doi: 10.4187/respcare.10644 37253612 PMC10468167

[12] Jayakumaran J, Smaldone GC. *In vivo* deposition of high-flow nasal aerosols using breath-enhanced nebulization. Pharmaceutics 2024;16(2):182; doi: 10.3390/pharmaceutics16020182 38399243 PMC10891871

[13] Devadason SG, Chan HK, Haeussermann S, et al. Validation of radiolabeling of drug formulations for aerosol deposition assessment of orally inhaled products. J Aerosol Med Pulm Drug Deliv 2012;25(S1):S-6–S-9; doi: 10.1089/jamp.2012.1Su3 23215848

[14] Martini V, Hinchcliffe M, Blackshaw E, et al. Distribution of droplets and immune responses after aerosol and intra-nasal delivery of influenza virus to the respiratory tract of pigs. Front Immunol 2020;11:594470; doi: 10.3389/fimmu.2020.594470 33193445 PMC7653178

[15] Samuel J, Smaldone GC. Maximizing deep lung deposition in healthy and fibrotic subjects during jet nebulization. J Aerosol Med Pulm Drug Deliv 2020;33(2):108–115; doi: 10.1089/jamp.2019.1552 31855492 PMC7133424

[16] Smaldone GCP, Bennett RJ, Messina WD, et al. Interpretation of “24 hour lung retention” in studies of mucociliary clearance. J Aerosol Med. 1988;1(1):11–20; doi: 10.1089/jam.1988.1.11

[17] Fleming JS. A technique for the absolute measurement of activity using a gamma camera and computer. Phys Med Biol 1979;24(1):176–180; doi: 10.1088/0031-9155/24/1/017 372956

[18] Fleming JS, Conway JH, Holgate ST, et al. Evaluation of the accuracy and precision of lung aerosol deposition measurements from planar radionuclide imaging using simulation. Phys Med Biol 1998;43(8):2423–2429; doi: 10.1088/0031-9155/43/8/033 9725617

[19] Bennett WD, Smaldone GC. Human variation in the peripheral air-space deposition of inhaled particles. J Appl Physiol (1985) 1985;62(4):1603–1610. 10.1152/jappl.1987.62.4.1603 3597231

[20] Smaldone GC, Messina MS. Enhancement of particle deposition by flow-limiting segments in humans. J Appl Physiol (1985) 1985;59(2):509–514. 4030603 10.1152/jappl.1985.59.2.509

[21] Li J, Gong L, Fink JB. The ratio of nasal cannula gas flow to patient inspiratory flow on trans-nasal pulmonary aerosol delivery for adults: An in vitro study. Pharmaceutics 2019;11(5):225; doi: 10.3390/pharmaceutics11050225 31083346 PMC6571744

